# Medical visits, antihypertensive prescriptions and medication adherence among newly diagnosed hypertensive patients in Korea

**DOI:** 10.1186/s12199-017-0619-6

**Published:** 2017-03-17

**Authors:** Hyoseon Jeong, Hyeongsu Kim, Kunsei Lee, Jung Hyun Lee, Hye Mi Ahn, Soon Ae Shin, Vitna Kim

**Affiliations:** 1 Department of Preventive Medicine, School of Medicine, Konkuk University, 1 Hwayang-dong, Gwangjin-gu, Seoul, 05029 South Korea; 2 Bigdata Steering Department, National Health Insurance Service, Wonju, South Korea; 3 Department of Dental Hygiene, Suwon Women’s University, Suwon, South Korea

**Keywords:** Hypertension, Mass screening, Medication possession ratio, Medication adherence

## Abstract

**Objectives:**

The objective of this study was to assess the antihypertensive medication adherence in patients who were newly diagnosed with hypertension in Korea.

**Methods:**

Study subjects were diagnosed with hypertension for the first time by the General Health Screening in 2012 and were 65,919. As indices, visiting rate to medical institution, the antihypertensive prescription rate, medication possession ratio and the rate of appropriate medication adherence were used. The qualification data, the General Health Screening data and the health insurance claims data were used.

**Resutls:**

Visiting rate to medical institution within one-year was 42.3%. Gender, age, family history of hypertension, smoking status, drinking frequency, insurance type, BMI, hypertension status, blood glucose level and LDL-cholesterol level were significant variables for visiting a medical institution. Of the study subjects who visited a medical institution, the antihypertensive prescription rate was 89.1%. Medication possession ratio was 70.9% and the rate of appropriate medication adherence was 60.6%. Age, family history of hypertension, smoking status, BMI level, hypertension level, blood glucose level, status, and LDL-cholesterol level were significant variables for the antihypertensive prescription and gender, age, family history of hypertension, smoking status, BMI, hypertension status, and the time of the first visit to a medical institution were significant variables for appropriate medication adherence.

**Conclusions:**

This study showed that the antihypertensive medication adherence in patients who were newly diagnosed with hypertension was not relatively high in Korea. National Health Insurance Service should support an environment in which medical institutions and those diagnosed with hypertension can fulfill their roles.

## Introduction

Hypertension is a primary risk factor of myocardial infarction and stroke [1], and 29.2% (28.8–29.7%) of the world’s population is predicted to have hypertension by 2025 [2]. From 1990 to 2013, the number of fatal cases of hypertensive heart disease increased by 74.1% worldwide [3]. In addition, 7.6 million premature deaths (~13.5% of the global total) and 92 million disability adjusted life years (DALYs; 6.0% of the global total) were attributed to high blood pressure [4]. In Korea, the death rate associated with hypertension in 2013 was 10.0 per 100000 people [5], and the prevalence rate of hypertension was estimated to be approximately 25.5%, with a total associated medical insurance expenditure of approximately 2.5 trillion won [6]. Despite the high social and economic burdens related to hypertension, the management levels for hypertension had an awareness rate of 65.9%, treatment rate of 60.7%, and control rate of 42.5% in 2013 [7]. Approaches to increase the awareness, treatment, or control rates for hypertension are in high demand, and it has been suggested that one of the most effective methods is to identify people with hypertension and treat them as soon as possible.

Likewise, hypertension has high prevalence rate and also it causes a lot of medical expenses. If patients who suffer from hypertension were not treated properly with medication, it would develop complications which cause actual dollars of costs [8]. Hypertensive patients could lower their blood pressure through medical treatment and improvement of their lifestyle. And continual pharmacological treatment for hypertension decreases the hospitalization rate and lowers the risk of complications, such as myocardial infarction and stroke [9]. The government has a responsibility to raise a medication adherence in order to prevent hypertension-induced complications and reduce medical expenditure. Therefore, it is extremely important to figure out the medication adherence. There were several studies that dealt with the rate of visiting a medical institution, the antihypertensive prescription rate medication possession ratio or medical adherence among hypertensive patients [10–12], but they had some limitation like small sample size or restricted study area except one study that covered all hypertensive patients were aged 30 years or more and had received at least one antihypertensive prescription in Taiwan [13]. Furthermore there were no studies which purport for measuring medication adherence in Korea.

The aim of this study were to assess the antihypertensive medication adherence and related factors and to evaluate the status of medical visit and antihypertensive prescription, which are the pre-stage of medication adherence, in order to accurately assess medication adherence among those who were newly diagnosed with hypertension by the General Health Screening (GHS) in Korea 2012. The results could be used as evidence-based data to establish new healthcare policies and strategies for an efficient hypertension management.

## Materials and methods

### Study population

**Fig. 1:**
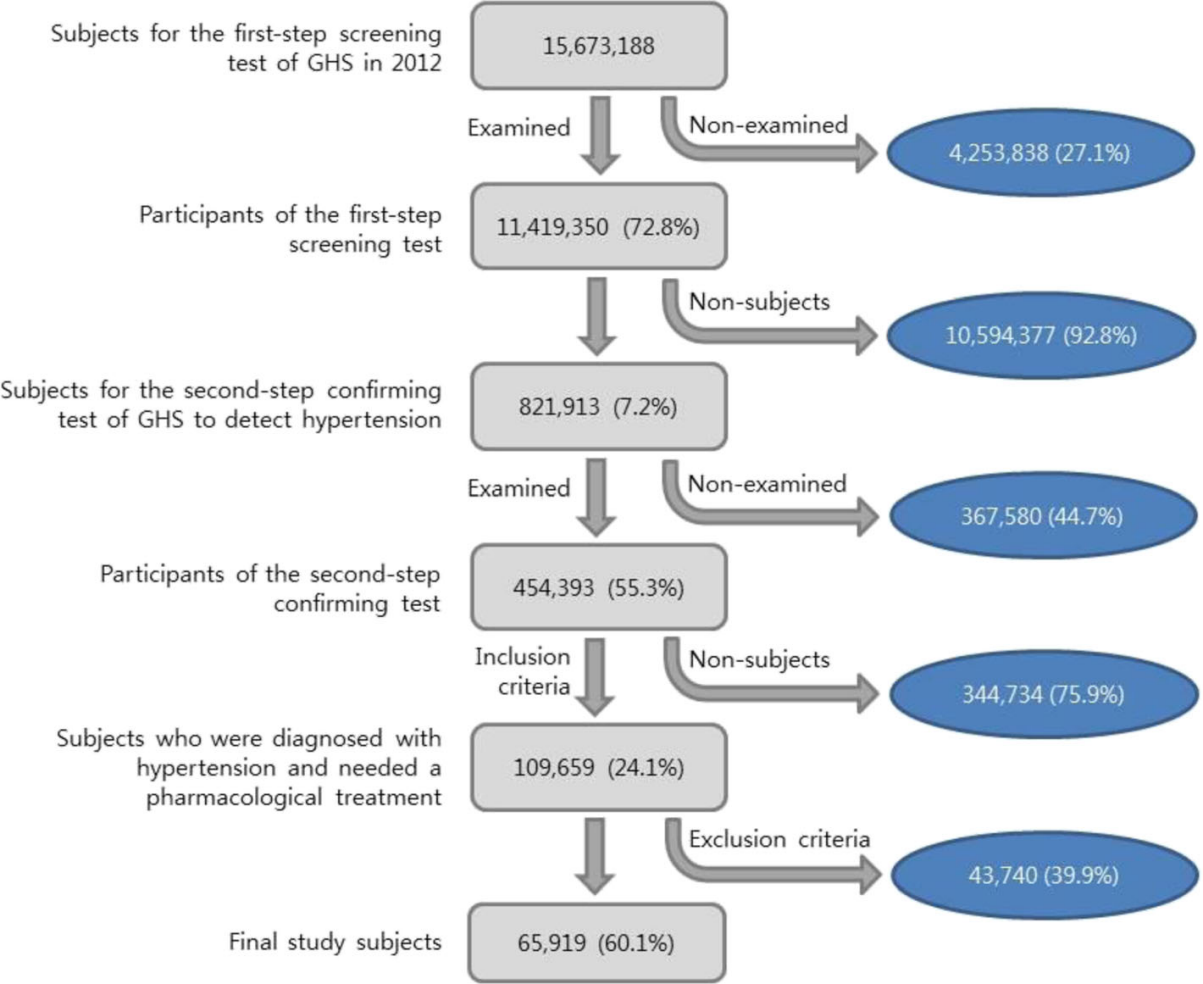
A selection process of the study subjects

The inclusion criteria for study subjects were as follows: 1) people who participated in the conducts GHS by the National Health Insurance Service (NHIS) in 2012; 2) participant who had blood pressure with more than 140 in systolic and 90 in diastolic at the first-step screen test and the second-step confirmatory test; 3) people who were diagnosed with hypertension in the result of GHS and were advised to have a pharmacological treatment to manage hypertension in the recommendation of GHS. The exclusion criteria were as follows: 1) participants who visited medical institutions due to hypertension, DM, dyslipidemia, myocardial infarction, stroke etc. as their principal or secondary diagnosis within the previous 3 years of the date of the second-step confirmatory test; 2) participants with a history of diagnosis and/or pharmacological treatment of hypertension, DM, dyslipidemia, myocardial infarction, stroke etc. based on the questionnaire of the first-step screening test; and 3) participants under 30 years of age at the time of the first-step screening test. The selection process of the final study subjects was shown in Fig. 1. The subjects for the first-step of GHS in 2002 were 15,673,188, of whom 11,419,350 (72.8%) completed the first-step screening test. Of these, 821,973 participants underwent the second-step confirmatory test of hypertension. Based on the results of the second-step confirmatory tests, 109,659 participants were diagnosed with hypertension and were advised to have a pharmacological treatment. Of these, 43,740 were excluded according to the exclusion criteria outlined above. Thus, the final study subjects of the present study were 65,919.

### Study data and variables

The present study used the qualification data, GHS data of 2012 and data of the insurance claims of medical institutions from January 2009 to December 2014 that were extracted from the NHIS administrative system. The qualification data were used to determine gender (male/female), age, and type of insurance policy; the GHS data were used to determine family history of hypertension, smoking status, alcohol drinking frequency, obesity status, hypertension status, blood glucose level, and blood low-density lipoprotein (LDL)-cholesterol level; and the insurance claim data were used to determine the time of the first visit to a medical institution and the number of days prescribed medication for hypertension.

The study subjects were categorized into various subgroups based on their demographic and clinical characteristics for the purposes of comparison. They were categorized based on age (every 10 years of age), insurance policy (regional, employment-based, and medical aid), smoking status (nonsmokers, ex-smokers, and smokers), and alcohol drinking frequency (nondrinking, once to twice per week, and more than three times per week). They were also categorized by obesity status according to body mass index (BMI) (normal: < 23 kg/m^2^, overweight: 23–24 kg/m^2^, obese: 25–29 kg/m^2^, and extremely obese:  ≥  30 kg/m^2^), hypertension level using data from the second-step screening (hypertension stage 1: 140–159 mmHg systolic pressure or 90–99 mmHg diastolic pressure, and hypertension stage 2: ≥ 160 mmHg systolic pressure or ≥  100 mmHg diastolic pressure), and blood glucose level using data from the first-step test (normal: < 126 mg/dL, mild: 126–139 mg/dL, moderate: 140–199 mg/dL, and severe: ≥ 200 mg/dL). Blood LDL-cholesterol levels (normal: < 130 mg/dL, mild: 130–159 mg/dL, severe: ≥ 160 mg/dL).

The subjects were further divided into subgroups according to the time of their first visit to a medical institution from the date of the second-step confirmatory test (≤ 90 days, 91–180 days, and 181–365 days).

### Measurement of major indices

#### Visiting rate to medical institution

A visit to a medical institution was used as an indicator of medical use. In this study, the visiting rate to medical institution was defined as the percentage of hypertensive patients who visited a medical institution as a principle or secondary diagnosis more than one time within 1 year

$$ \mathrm{Visiting}\ \mathrm{rate}\ \mathrm{t}\mathrm{o}\ \mathrm{medical}\ \mathrm{institution}=\frac{\mathrm{Number}\ \mathrm{o}\mathrm{f}\ \mathrm{hypertensive}\ \mathrm{patients}\ \mathrm{who}\ \mathrm{visited}\ \mathrm{a}\ \mathrm{medical}\ \mathrm{institution}\ \mathrm{within}\ 1\ \mathrm{year}\ \mathrm{f}\mathrm{rom}\ \mathrm{t}\mathrm{he}\ \mathrm{date}\ \mathrm{a}\mathrm{f}\mathrm{t}\mathrm{er}\ \mathrm{diagnosis}}{\mathrm{Final}\ \mathrm{number}\ \mathrm{o}\mathrm{f}\ \mathrm{study}\ \mathrm{subjects}}\times 100 $$

#### Antihypertensive prescription rate

The antihypertensive prescription rate was calculated as the percentage of subjects who received an antihypertensive prescription among the subjects who visited a medical institution.

$$ \mathrm{Antihypertensive}\ \mathrm{prescription}\ \mathrm{rate}=\frac{\mathrm{Number}\ \mathrm{of}\ \mathrm{subjects}\ \mathrm{receiving}\ \mathrm{a}\mathrm{n}\ \mathrm{a}\mathrm{n}\mathrm{tihypertensive}\ \mathrm{prescription}}{\mathrm{Number}\ \mathrm{of}\ \mathrm{study}\ \mathrm{subjects}\ \mathrm{who}\ \mathrm{visited}\ \mathrm{a}\ \mathrm{medical}\ \mathrm{institution}}\times 100 $$

#### Medication possession ratio (MPR)

The MPR was defined as the percentage of the sum of days prescribed antihypertensive medication within 1 year from the first prescribed day among the subjects who visited a medical institution over the 365-day period [14, 15]. However, we operationally defined MPR as the percentage of sum of the purchased days of antihypertensive medication within 1 year from the first day of purchasing prescribed medication in this study.

$$ \mathrm{Medication}\ \mathrm{possession}\ \mathrm{ratio}=\frac{\mathrm{Sum}\ \mathrm{of}\ \mathrm{the}\ \mathrm{purchased}\ \mathrm{day}\mathrm{s}\ \mathrm{of}\ \mathrm{antihypertensive}\ \mathrm{medication}\ \mathrm{within}\ \mathrm{the}\ \mathrm{period}\ \mathrm{of}\ \mathrm{denominator}\ \left(\mathrm{one}\ \mathrm{year}\right)}{\mathrm{One}\ \mathrm{year}\ \mathrm{from}\ \mathrm{the}\ \mathrm{first}\ \mathrm{day}\ \mathrm{of}\ \mathrm{purchasing}\ \mathrm{prescribed}\ \mathrm{medication}\ \left(365\ \mathrm{day}\mathrm{s}\right)}\times 100 $$

When the MPR was greater than 100%, it was adjusted to 100%.

#### Rate of appropriate medication adherence

Appropriate medication adherence (AMA) was defined as the value of MPR greater than 80% [16, 17]. The rate of AMA was calculated as the percentage of subjects who had a MPR ≥  80% among those who purchased an antihypertensive medication based on the prescription by a physician.

$$ \mathrm{Rate}\ \mathrm{of}\ \mathrm{appropriate}\ \mathrm{medication}\ \mathrm{adherence}=\frac{\mathrm{Number}\ \mathrm{of}\ \mathrm{subjects}\ \mathrm{with}\ \mathrm{medication}\ \mathrm{possession}\ \mathrm{ratio}\ \ge\ 80\%}{\mathrm{Number}\ \mathrm{of}\ \mathrm{subjects}\ \mathrm{who}\ \mathrm{purchased}\ \mathrm{an}\ \mathrm{an}\mathrm{tihypertensive}\ \mathrm{medication}}\times 100 $$

### Data analysis

The chi-square test, *t* test, and analysis of variance were used for comparisons within subgroups of the following variables: the rate of visiting a medical institution, the antihypertensive prescription rate, MPR and rate of AMA, respectively. Next, multivariate logistic regression analysis was performed to identify the variables significantly related to visiting a medical institution, antihypertensive prescription rate, and rate of AMA. The odds ratios (OR) and 95% confidence intervals (CI) of the visiting rate were calculated. Statistical analyses in the present study were conducted using SAS software (version 9.1; SAS Institute Inc., Cary, NC, USA). In all analyses, *p*  <  0.05 was taken to indicate statistical significance.

## Results

### Visiting rate to medical institution and its related factors

The rate of visiting a medical institution was 42.3% (*n* = 27,895), 37.9% for males, and 56.3% for females (*p* < 0.001). The rates of visiting a medical institution according to various variables are shown in Table 1. Based on the results of multivariate logistic regression analysis, the variables significantly associated with visiting a medical institution for hypertension treatment were gender, age, family history of hypertension, smoking status, drinking frequency, insurance type, BMI, hypertension status, blood glucose level and LDL-cholesterol level. The OR and 95% CI for the rate of visiting a medical institution for hypertension according to the variables evaluated are shown in Table 2.

**Table 1:** Distribution of visiting rates to medical institutions according to the variables

Variables		Medical institution		Total		*P*-value
		Nonvisiting (%)	Visiting (%)			
Total		38,024 (57.7)	27,895 (42.3)	65,919	(100)	
Gender	Male	31,068 (62.1)	18,927 (37.9)	49,995	(75.8)	<0.001
	Female	6,956 (43.7)	8,968 (56.3)	15,924	(24.2)	
Age group	30–39 years	10,785 (79.1)	2,855 (20.9)	13,640	(20.7)	<0.001
	40–49 years	12,898 (61.2)	8,187 (38.8)	21,085	(31.9)	
	50–59 years	9,817 (49.9)	9,877 (50.1)	19,694	(29.9)	
	60–69 years	3,432 (40.4)	5,055 (59.6)	8,487	(12.9)	
	≥70 year	1,092 (36.2)	1,921 (63.8)	3,013	(4.6)	
Family history of hypertension	Not present	26,112 (64.5)	14,400 (35.5)	40,512	(81.7)	<0.001
	Present	4,990 (55.1)	4,059 (44.9)	9,049	(18.3)	
Smoking status	Nonsmoker	14,389 (51.6)	13,517 (48.4)	27,906	(42.4)	<0.001
	Ex-smoker	8,325 (56.8)	6,335 (43.2)	14,660	(22.2)	
	Smoker	15,295 (65.6)	8,032 (34.4)	23,327	(35.4)	
Drinking frequency (per week)	Nondrinking	10,867 (49.1)	11,250 (50.9)	22,117	(34.4)	<0.001
	1–2 times	17,585 (64.3)	9,775 (35.7)	27,360	(42.5)	
	More than 3 times	8,845 (59.3)	6,074 (40.7)	14,919	(23.1)	
Insurance type	Regional	4,495 (38.6)	7,159 (61.4)	11,654	(17.7)	<0.001
	Employment-based	33,411 (61.9)	20,547 (38.1)	53,958	(81.8)	
	Medical aid	118 (38.4)	189 (61.6)	307	(0.5)	
BMI level	Normal	8,595 (56.6)	6,585 (43.4)	15,180	(23.0)	<0.001
	Overweight	9,027 (56.1)	7,061 (43.9)	16,088	(24.4)	
	Obese	16,373 (57.9)	11,903 (42.1)	28,276	(42.9)	
	Extremely obese	4,026 (63.2)	2,345 (36.8)	6,371	(9.7)	
Hypertension level	Hypertension stage 1	19,749 (62.9)	11,664 (37.1)	31,413	(47.7)	<0.001
	Hypertension stage 2	18,272 (53.0)	16,230 (47.0)	34,502	(52.3)	
Blood glucose level	Normal	35,742 (58.3)	25,570 (41.7)	61,312	(93.0)	<0.001
	Mild	1,109 (53.9)	949 (46.1)	2,058	(3.1)	
	Moderate	903 (48.4)	964 (51.6)	1,867	(2.9)	
	Severe	265 (39.1)	412 (60.9)	677	(1.0)	
Blood LDL level	Normal	23,781 (59.8)	16,000 (40.2)	39,781	(61.5)	<0.001
	Mild	9,127 (55.9)	7,214 (44.1)	16,341	(25.3)	
	Severe	4,348 (50.9)	4,188 (49.1)	8,536	(13.2)	

Data are expressed as the number (%)

*P*-value from chi-square test for binary outcomes comparing a difference between of nonvisiting and visiting

**Table 2:** Factors associated with visits to medical institutions according to multiple logistic regression analysis

		Visit to medical institution	
		OR^a^	95% CI^b^
Gender	Male	1	
	Female	1.57	1.48–1.67
Age group	30–39 years	1	
	40–49 years	2.05	1.93–2.18
	50–59 years	3.28	3.07–3.49
	60–69 years	4.81	4.43–5.22
	≥70 year	6.37	5.63–7.22
Family history of hypertension	Not present	1	
	Present	1.58	1.50–1.66
Smoking status	Nonsmoker	1.10	1.03–1.16
	Ex-smoker	1.26	1.19–1.33
	Smoker	1	
Drinking frequency (per week)	Nondrinking	0.98	0.92–1.04
	1–2 times	0.89	0.85–0.94
	More than 3 times	1	
Insurance type	Regional	1.85	1.75–1.96
	Employment-based	1	
	Medical aid	2.11	1.51–2.96
BMI level	Normal	1	
	Overweight	1.14	1.07–1.20
	Obese	1.17	1.11–1.23
	Extremely obese	1.19	1.10–1.29
Hypertension level	Hypertension stage 1	1	
	Hypertension stage 2	1.86	1.78–1.94
Blood glucose level	Normal	1	
	Mild	1.11	0.99–1.25
	Moderate	1.47	1.30–1.66
	Severe	2.19	1.78–2.70
Blood LDL level	Normal	1.	
	Mild	1.08	1.03–1.13
	Severe	1.17	1.10–1.24

^a^ *OR* odds ratio, ^b^ *CI* confidence interval

### Antihypertensive prescription rate and its related factors

Of the subjects who visited a medical institution, the antihypertensive prescription rate was 89.1% (*n* = 24,861). The antihypertensive prescription rates according to the variables evaluated are shown in Table 3. Based on the results of multivariate logistic regression analysis, the variables significantly associated with antihypertensive prescription rate were age, family history of hypertension, smoking status, BMI level, hypertension level, blood glucose level, status, and LDL-cholesterol level. The OR and 95% CI for the antihypertensive prescription rate according to the variables evaluated are shown in Table 4.

**Table 3:** Distribution of antihypertensive prescription rates according to the variables

Variables		Antihypertensive prescription		Total	*P*-value
		Not receiving (%)	Receiving (%)		
Total		3,033 (10.9)	24,861 (89.1)	27,894 (100)	
Gender	Male	1,994 (10.5)	16,933 (89.5)	18,927 (67.9)	0.009
	Female	1,039 (11.6)	7,928 (88.4)	8,967 (32.1)	
Age group	30–39 years	415 (14.5)	2,440 (85.5)	2,855 (10.2)	<0.001
	40–49 years	781 (9.5)	7,406 (90.5)	8,187 (29.4)	
	50–59 years	1,000 (10.1)	8,877 (89.9)	9,877 (35.4)	
	60–69 years	594 (11.8)	4,461 (88.2)	5,055 (18.1)	
	≥70 year	243 (12.7)	1,677 (87.3)	1,920 (6.9)	
Family history of hypertension	Not present	1,615 (11.2)	12,784 (88.8)	14,399 (78.0)	0.057
	Present	412 (10.2)	3,647 (89.8)	4,059 (22.0)	
Smoking status	Nonsmoker	1,568 (11.6)	11,984 (88.4)	13,516 (48.5)	<0.001
	Ex-smoker	699 (11.0)	5,636 (89.0)	6,335 (22.7)	
	Smoker	765 (9.5)	7,267 (90.5)	8,032 (28.8)	
Drinking frequency (per week)	Nondrinking	1,314 (11.7)	9,935 (88.3)	11,249 (41.5)	<0.001
	1–2 times	1,084 (11.1)	8,691 (88.9)	9,775 (36.1)	
	More than 3 times	569 (9.4)	5,505 (90.6)	6,074 (22.4)	
Insurance type	Regional	757 (10.6)	6,402 (89.4)	7,159 (25.7)	0.435
	Employment-based	2,259 (11.0)	18,287 (89.0)	20,546 (73.6)	
	Medical aid	17 (9.0)	172 (91.0)	189 (0.7)	
BMI level	Normal	789 (12.0)	5,796 (88.0)	6,585 (23.6)	<0.001
	Overweight	811 (11.5)	6,250 (88.5)	7,061 (25.3)	
	Obese	1,228 (10.3)	10,608 (89.7)	11,902 (42.7)	
	Extremely obese	205 (8.7)	2,090 (91.3)	2,354 (8.4)	
Hypertension level	Hypertension stage 1	1,620 (13.9)	10,043 (86.1)	11,663 (41.8)	<0.001
	Hypertension stage 2	1,413 (8.7)	14,817 (91.3)	16,230 (58.2)	
Blood glucose level	Normal	2,820 (11.0)	22,749 (89.0)	25,569 (91.7)	0.007
	Mild	97 (10.2)	852 (89.8)	949 (3.3)	
	Moderate	90 (9.3)	874 (90.7)	964 (3.5)	
	Severe	26 (6.3)	386 (93.7)	412 (1.5)	
Blood LDL level	Normal	1,603 (10.0)	14,397 (90.0)	16,000 (58.4)	<0.001
	Mild	820 (11.4)	6,394 (88.6)	7,214 (26.3)	
	Severe	580 (13.8)	3,607 (86.2)	4,187 (15.3)	

Data are expressed as the number (%)

*P*-value from chi-square test for binary outcomes comparing a difference between of not receiving prescription and receiving prescription

**Table 4:** Factors associated with antihypertensive prescription according to multiple logistic regression analysis

		Antihypertensive prescription	
		OR^a^	95% CI^b^
Gender	Male	1	
	Female	1.03	0.89–1.18
Age group	30–39 years	1	
	40–49 years	1.83	1.56–2.13
	50–59 years	1.92	1.64–2.25
	60–69 years	1.82	1.52–2.19
	≥70 year	1.50	1.18–1.90
Family history of hypertension	Not present	1	
	Present	1.15	1.02–1.30
Smoking status	Nonsmoker	0.78	0.68–0.90
	Ex-smoker	0.86	0.75–0.99
	Smoker	1	
Drinking frequency (per week)	Nondrinking	0.94	0.81–1.09
	1–2 times	0.87	0.76–1.00
	More than 3 times	1	
Insurance type	Regional	0.93	0.82–1.04
	Employment-based	1	
	Medical aid	1.16	0.58–2.31
BMI level	Normal	1	
	Overweight	1.12	0.98–1.28
	Obese	1.32	1.17–1.49
	Extremely obese	1.69	1.37–2.07
Hypertension level	Hypertension stage 1	1	
	Hypertension stage 2	1.72	1.56–1.89
Blood glucose level	Normal	1	
	Mild	0.87	0.67–1.13
	Moderate	0.99	0.75–1.31
	Severe	1.86	1.08–3.22
Blood LDL level	Normal	1	
	Mild	0.82	0.73–0.91
	Severe	0.66	0.58–0.76

^a^ *OR* odds ratio, ^b^ *CI* confidence interval

### MPR, rate of AMA, and its related factors

Of the subjects who received an antihypertensive prescription, the subjects who purchased antihypertensive medication were 24,449 (98.3%). Among them, the MPR was 70.9%, the rate of AMA was 60.6%, and the MPR and rate of AMA by the variables evaluated are shown in Table 5. Based on the results of multivariate logistic regression analysis, the variables significantly associated with AMA were gender, age, family history of hypertension, smoking status, BMI, hypertension status, and the time of the first visit to a medical institution. The OR and 95% CI for the rate of AMA according to the variables evaluated are shown in Table 6.

**Table 5:** Distribution of the medication possession ratio and appropriate medication adherence according to the variables

Variables		Medication possession ratio		Appropriate medication adherence			
		Mean (SD)	*P*-value	MPR < 80%	MPR ≥ 80%	Total	*P*-value
Total		70.9 (36.4)		9,640 (39.4)	14,809 (60.6)	24,449 (100)	
Gender	Male	69.1 (37.1)	<0.001	6,919 (41.6)	9,713 (58.4)	16,632 (68.0)	<0.001
	Female	74.7 (34.5)		2,721 (34.8)	5,096 (65.2)	7,817 (32.0)	
Age group	30–39 years	57.9 (39.3)	<0.001	1,306 (54.9)	1,071 (45.1)	2,377 (9.7)	<0.001
	40–49 years	70.4 (36.2)		2,939 (40.4)	4,342 (59.6)	7,281 (29.8)	
	50–59 years	73.2 (35.4)		3,180 (36.3)	5,577 (63.7)	8,757 (35.8)	
	60–69 years	74.2 (35.1)		1,555 (35.5)	2,832 (64.5)	4,387 (17.9)	
	≥70 year	69.8 (37.5)		660 (40.1)	987 (59.9)	1,647 (6.7)	
Family history of hypertension	Not present	71.7 (35.9)	<0.001	4,870 (38.7)	7,716 (61.3)	12,586 (77.8)	<0.001
	Present	74.2 (34.8)		1,259 (35.0)	2,336 (65.0)	3,595 (22.2)	
Smoking status	Nonsmoker	72.6 (35.6)	<0.001	4,415 (37.5)	7,358 (62.5)	11,773 (48.2)	<0.001
	Ex-smoker	73.2 (35.6)		2,003 (36.2)	3,534 (63.8)	5,537 (22.7)	
	Smoker	66.2 (37.9)		3,220 (45.2)	3,909 (54.8)	7,129 (29.2)	
Drinking frequency (per week)	Nondrinking	73.1 (35.4)	<.0001	3,578 (36.6)	6,209 (63.4)	9,787 (41.2)	<0.001
	1–2 times	69.6 (36.9)		3,495 (40.9)	5,042 (59.1)	8,537 (36.0)	
	More than 3 times	69.3 (36.9)		2,248 (41.6)	3,163 (58.4)	5,411 (22.8)	
Insurance type	Regional	70.5 (36.4)	0.340	2,512 (39.9)	3,791 (60.1)	6,303 (25.8)	0.504
	Employment-based	71.0 (36.4)		7,055 (39.3)	10,919 (60.7)	17,974 (73.5)	
	Medical aid	70.3 (34.7)		73 (42.4)	99 (57.6)	172 (0.7)	
BMI level	Normal	69.7 (36.9)	<0.001	2,341 (41.0)	3,367 (59.0)	5,708 (23.4)	<0.001
	Overweight	71.9 (36.1)		2,341 (38.0)	3,811 (62.0)	6,152 (25.2)	
	Obese	71.8 (35.9)		4,036 (38.5)	6,461 (61.5)	10,497 (42.9)	
	Extremely obese	66.4 (37.8)		922 (44.1)	1,169 (55.9)	2,091 (8.6)	
Hypertension level	Hypertension stage 1	69.3 (37.1)	<0.001	4,052 (41.1)	5,809 (58.9)	9,861 (40.3)	<0.001
	Hypertension stage 2	71.9 (35.8)		5,587 (38.3)	9,000 (61.7)	14,587 (59.7)	
Blood glucose level	Normal	70.9 (36.4)	0.228	8,814 (39.4)	13,554 (60.6)	22,368 (91.5)	0.670
	Mild	69.9 (37.2)		331 (39.3)	511 (60.7)	842 (3.4)	
	Moderate	69.6 (36.5)		351 (41.0)	504 (59.0)	855 (3.5)	
	Severe	73.9 (33.9)		144 (37.5)	240 (62.5)	384 (1.6)	
Blood LDL level	Normal	70.6 (36.6)	0.355	5,590 (39.5)	8,547 (60.5)	14,137 (58.9)	0.716
	Mild	71.3 (36.2)		2,474 (39.3)	3,828 (60.7)	6,302 (26.3)	
	Severe	71.4 (35.9)		1,381 (38.8)	2,177 (61.2)	3,558 (14.8)	
Time of the first visit to a medical institution (days)	Within 90	69.7 (37.0)	0.301	7,624 (40.7)	11,108 (59.3)	18,732 (76.6)	<0.001
	91–180	75.1 (33.7)		779 (34.6)	1,474 (65.4)	2,253 (9.2)	
	After 181	74.5 (34.2)		1,237 (35.7)	2,227 (64.3)	3,464 (14.2)	

Medication possession ratio's data are expressed as the mean (SD)

Appropriate medication adherence’s data are expressed as the number (%)

*P*-value from chi-square test for binary outcomes comparing a difference between of MPR ≥ 80% and MPR < 80%

**Table 6:** Factors associated with the appropriate medication adherence according to multiple logistic regression analysis

		Appropriate medication adherence	
		OR^a^	95% CI^b^
Gender	Male	1	
	Female	1.22	1.10–1.34
Age group	30–39 years	1	
	40–49 years	1.72	1.53–1.92
	50–59 years	2.17	1.93–2.43
	60–69 years	2.28	1.99–2.62
	≥70 year	2.00	1.66–2.41
Family history of hypertension	Not present	1	
	Present	1.26	1.16–1.37
Smoking status	Nonsmoker	1.07	0.97–1.18
	Ex-smoker	1.30	1.19–1.43
	Smoker	1	
Drinking frequency (per week)	Nondrinking	1.11	1.00–1.23
	1–2 times	1.05	0.96–1.14
	More than 3 times	1	
Insurance type	Regional	0.93	0.86–1.01
	Employment-based	1	
	Medical aid	0.78	0.50–1.19
BMI level	Normal	1	
	Overweight	1.12	1.01–1.23
	Obese	1.14	1.04–1.25
	Extremely obese	1.03	0.90–1.18
Blood pressure level	Hypertension stage 1	1	
	Hypertension stage 2	1.24	1.16–1.33
Blood glucose level	Normal	1	
	Mild	0.89	0.74–1.07
	Moderate	0.94	0.78–1.14
	Severe	1.24	0.93–1.66
Blood LDL level	Normal	1	
	Mild	0.91	0.84–0.98
	Severe	0.96	0.87–1.06
Time of the first visit to a medical institution (days)	Within 90	1	
	91–180	1.29	1.15–1.44
	After 181	1.32	1.20–1.44

^a^ *OR* odds ratio, ^b^ *CI* confidence interval

## Discussion

This study was performed to assess the antihypertensive medication adherence in patients who were newly diagnosed with hypertension by the 2012 GHS conducted by the NHIS on commencement of pharmacological treatment and sustainability of antihypertensive medication adherence using the followings indices: rate of visiting a medical institution within 1 year after the date of the second-step confirmatory test, antihypertensive prescription rate within 1 year after the first visit, MPR, and rate of AMA.

For effective management of hypertension, it is imperative to receive an early diagnosis and to visit a medical institution for pharmacological treatment as well as nonpharmacological management by a physician.

First, the rate of visiting a medical institution by the study subjects diagnosed with hypertension was 42.3% in this study. In addition, the rate of visiting a medical institution was 20.9% for study subjects in their 30s. These results could be interpreted as the early finding of chronic disease such as hypertension by GHS did not induce the early treatment effectively. These results were related with the fact that the rates of awareness and treatment for hypertension in the 30s in Korea were 19.1 and 12.4% [18]. In order to manage hypertension and prevent its complications, first of all, there is a need to alert those with risk factors for hypertension and those diagnosed but who not visit a medical institution to improve the awareness rate. In addition, the rate of visiting a medical institution within 1 year of diagnosis was higher in women than men, those of advance age, those with a family history of hypertension, ex-smokers, those with a high BMI, those with more severe hypertension, or those with other chronic diseases, such as diabetes or hyperlipidemia.

Next, among subjects who visited a medical institution, 89.1% received the antihypertensive prescription by a physician. The antihypertensive prescription rate was low among relatively healthy subjects, i.e., those in their 30s with no family history of hypertension, and those with a normal BMI. The guidelines of JNC-8 and European Society of Hypertension address treatment according to hypertension level and risk factors [19, 20]. Lifestyle changes are primarily recommended for those in low- or medium-risk groups. Based on these guidelines, physicians often recommended lifestyle changes instead of antihypertensive medication for subjects who were relatively healthy and belong to the low-risk group.

Finally, among subjects who received the antihypertensive prescription, 98.3% purchased antihypertensive medication. Among them, the MPR was 70.9% and the rate of AMA was 60.6%. Medication adherence of women (62.5%) was better than that of men (58.4%), that of advanced age (≥70: 59.9%), that with a relevant family history, that with a high BMI, that with high blood pressure, and that with a delayed first visit to a medical institution. There were some studies about medication adherence for hypertension. For example, Rolnick et al. revealed that medication adherence was better in men (70.5%) than in women (68.8%), those of advanced age (≥70: 70.5%) in the U.S, [10]. Yang et al. reported that medication adherence was 43.5% and better in men (47.2%) than in women (40.3%), those of advanced age (≥70: 55.6%), those with having a knowledge about hypertension needs lifelong medical treatment (47.1%) in Beijing [11]. And Inkster et al. found that medication adherence was better in men (87%) than in women (85%), those of a advanced age (≥70: 91%), those with high comorbidities (2+: 91%) in the UK [12]. Lee et al. showed that approximately 53% of the patients had high compliance with antihypertensive medication in Taiwan [13]. Since each study and disease has its own definitions of the evaluation methods, medication adherence, and follow-up periods for medication adherence, it is not easy to compare the results directly among studies. Nevertheless, the MPR for hypertension treatment in this study was higher than that of Taiwan but lower than those of western countries. Meanwhile, age and comorbidity were known as an important factors related to expectations regarding treatment, and attitude to taking medication [21, 22]. Similarly, the rates of visiting a medical institution and of AMA were low among younger subjects and some subgroups, such as subjects who were overweight or had higher LDL-cholesterol levels, had higher rates of AMA in this study. The rate of medication adherence was also high when the satisfaction rate regarding medication counseling was high [23], while “forgetting to take the medication” was the principal reason for decreased medication adherence [24]. According to a previous systematic review study regarding the MPR and the rate of AMA for chronic diseases [25], 12-month MPR was 67% and the rate of AMA was 64% in hypertension, MPR was 76% and the rate of AMA was 58% in diabetes, and MPR was 74% and the rate of AMA was 51% in dyslipidemia. To improve medication adherence, it is necessary for a physician to promote and educate hypertension patients themselves to recognize the importance of medication treatment, and to provide appropriate counseling services them to change the health behaviors like smoking, physical activities etc.

This study had several limitations. First, as the data from GHS and health insurance claims were used, this study had the limitation of using secondary data. Therefore, we could not examine the characteristics of the study population, such as knowledge, attitude, or access to medical institutions, etc. Second, this study dealt only with a study population with primary or secondary disease codes for hypertension. Therefore, it is likely that some data were missing, because they were not claimed as primary or secondary disease codes. However, the chance of this was very low because the study population was diagnosed with hypertension for the first time through GHS in 2012. Third, medication adherence was determined as the MPR based on the purchase of antihypertension medication that was prescribed by a physician. MPR cannot be used to verify the intake of the medications. However, the prescription is significant as the first step in taking a medication, and there have been reports that the MPR that use the prescription is a good index for verifying medication adherence [26]. Furthermore, it is almost impossible to verify medication compliance in studies based on massive databases. In order to overcome the limitation of prescription that can not verify medication compliance, we used the purchase of antihypertension medication that is the next step after receiving the antihypertensive prescription. Of the subjects who received an antihypertensive prescription, the rate of the purchase of antihypertensive medication was 98.3% in this study. MPR and the rate of AMA based on the prescription were 69.9 and 59.7% (not described in the result) but those of based on the purchase were 70.9 and 60.6%. We could find there was the difference values of MPR and the rate of AMA between the data. In fact, patients who purchased the medication take more positive behavior on treatment than patients who were just prescribed medication.

This study showed that the antihypertensive medication adherence in patients who were newly diagnosed with hypertension was not relatively high in Korea. First of all, those diagnosed with hypertension should visit a medical institution to increase the MPR and rate of AMA. Medical institutions that diagnose hypertension should notify and educate hypertensive patients of their medical situation and encourage them to participate actively in treatment. Next, those diagnosed with hypertension should follow the directions of their physicians and cooperate to manage their hypertension. NHIS should make and support an environment in which medical institutions and those diagnosed with hypertension can fulfill their roles.
